# Editorial 2022

**DOI:** 10.1098/rsos.220229

**Published:** 2022-03-09

**Authors:** Wendy Hall

**Affiliations:** Web Science Institute, University of Southampton, Southampton, Hampshire, UK

I join *Royal Society Open Science* as the second postholder as the Editor-in-Chief for the journal, and I would like to start not only by thanking my predecessor Prof. Jeremy Sanders CBE FRS and the members of the Editorial Board for their hard work to this point but also congratulating them on developing such an interesting journal. I was delighted when the Royal Society launched *Royal Society Open Science* in 2014 and have closely followed its progress since then in my role as Chair of the Publications Board. The journal has established itself as a high-quality open access publication, and a venue allowing the Society to experiment with open peer review (versions of which have subsequently been adopted by a number of other Royal Society journals), pre-registration of studies (in the form of Registered Reports), Replication studies and last year—under the difficult circumstances with which we are all familiar—it launched a new section ‘Science, Society and Policy’, an exciting innovation and one I look forward to developing further.

My research interests lie in computer science, but span several disciplinary boundaries, including governance and policy with respect to artificial intelligence and data governance and the Internet—both areas that will have huge significance for society and science in future. *Royal Society Open Science* is an avowedly cross-disciplinary journal and this is a further source of interest for me. I see a lot of opportunities to encourage more cross-disciplinary work, and the journal aims to publish a special collection later this year that showcases ground-breaking research and high-quality reviews and perspectives that explore artificial intelligence from a cross-disciplinary perspective. This collection has been jointly commissioned by a number of the Subject Editors and I am looking forward to reading content in this collection. The journal has also previously published ‘New talent’ collections of work commissioned from holders of Royal Society grants (such as University Research Fellows and Dorothy Hodgkin Fellowship holders), an initiative that I would like to continue in other areas of the journal, particularly those that have historically not been as well represented in terms of submitted manuscripts as might be expected.

On the subject of representation, I have long been interested in broadening access to science, not only in terms of open access and open data but in encouraging greater diversity in the publishing (and wider scientific) landscape. My new role will allow me to do more in the journal space to encourage diversity, and I will be working closely with the journal administrative office and Royal Society to improve diverse representation in our author base, our reviewers and our Editorial Board. I'm particularly pleased that Prof Sanders has taken on the Chair for the Society's Diversity Committee, and I plan to seek his guidance on how to further support under-represented groups in *Royal Society Open Science*.

*Royal Society Open Science* is certainly a successful journal, and with the strong foundations laid by my predecessor and our colleagues on the Editorial Board (working the Society's in-house teams), I am looking forward to taking the journal to greater heights and future successes. I hope you will work with and join me on this exciting journey.

